# Pulmonary rehabilitation in post-tuberculosis lung disease: a systematic review

**DOI:** 10.3389/fmed.2026.1929171

**Published:** 2026-08-19

**Authors:** Kishore Kumar, Adhishree Chidambaram, Manigandan Venkatesan, Husam I. Alahmadi, Ghazi A. Alotaibi, Hajed M. Al-Otaibi

**Affiliations:** 1 Department of Respiratory Therapy, Batterjee Medical College, Jeddah, Saudi Arabia; 2 The University of Texas at San Antonio, San Antonio, TX, United States; 3 Department of Medicine, University of Texas Health Science Centre, San Antonio, TX, United States; 4 Department of Respiratory Therapy, Faculty of Medical Rehabilitation Sciences, King Abdulaziz University, Jeddah, Saudi Arabia; 5 Respiratory Therapy Unit, King Abdulaziz University Hospital, King Abdulaziz University, Jeddah, Saudi Arabia; 6 Department of Respiratory Care, College of Applied Medical Sciences, Imam Abdulrahman Bin Faisal University, Dammam, Saudi Arabia; 7 College of Medicine and Health Sciences, Arabian Gulf University, Manama, Bahrain

**Keywords:** post-tuberculosis, PTLD, pulmonary rehabilitation, respiratory rehabilitation, tuberculosis

## Abstract

**Objective:**

Tuberculosis (TB) remains a significant global health concern, resulting in illness and death for millions of individuals. Despite the availability of effective treatment options, a substantial number of people continue to suffer from long-term lung consequences known as post-tuberculosis lung disease (PTLD). PTLD may be associated with reduced exercise capacity, respiratory impairment, and diminished quality of life. Pulmonary rehabilitation (PR), an intervention that combines exercise and education to enhance pulmonary function and overall wellbeing, may offer a solution. The aim of this systematic review was to map the available evidence on PR interventions for PTLD, identify gaps in the literature, and synthesize findings on exercise capacity, quality of life, pulmonary function, and symptom outcomes to inform clinical practice and future research.

**Methods:**

A systematic search was conducted across four electronic databases, Scopus, PubMed (MEDLINE), ProQuest, and EBSCO, for studies published between 2000 and 2026. The overall search yielded 1,085 articles. After reviewing titles and abstracts, we removed 163 duplicates and excluded 905 articles. A total of 17 articles met the inclusion criteria and were included in this systematic review.

**Results:**

Seventeen studies met the inclusion criteria, encompassing randomized controlled trials (RCTs) and observational study designs across multiple countries. PR consistently improved exercise capacity, with a 6 min walk distance (6MWD) increasing by 36–189 m (440.6 ± 70.8 m to 574.6 ± 168.9 m; 399 ± 62 m to 467 ± 65 m, *p* < 0.01) and a significant improvement in quality of life, as evidenced by reductions in St Georges Respiratory Questionnaire (SGRQ) scores of up to 13 points (*p* < 0.05) and significant between-group differences in some studies (*p* < 0.0001). Symptom outcomes also improved, including reductions in dyspnea and chest pain (44.8% to 24.1%). Pulmonary function findings were heterogeneous; some studies reported significant improvements in FEV_1_ (2.94 ± 0.68 L to 3.18 ± 0.54 L, *p* < 0.001), whereas others showed no significant changes.

**Conclusion:**

Despite certain limitations, all included studies demonstrated improvement in at least one key outcome domain. They collectively suggest that PR may be a valuable component of PTLD management, though further high-quality trials are needed to establish optimal protocols and confirm efficacy. PR has the potential to enhance the quality of life, exercise capacity, and lung function in patients with post-TB complications.

## Introduction

According to the World Health Organization (WHO), an estimated 10.7 million people developed tuberculosis (TB) globally in 2024, and approximately 1.23 million died from the disease. TB remains one of the top 10 causes of death worldwide and continues to be the leading cause of death from a single infectious agent (1). Despite being a preventable and treatable disease, TB continues to pose a major global public health challenge. Nevertheless, significant progress has been achieved through effective diagnosis and treatment strategies, with an estimated 83 million deaths averted worldwide between 2000 and 2024 owing to TB interventions (1). The significance of post-tuberculosis lung disease (PTLD) as a contributor to chronic respiratory diseases (CRD) has been largely overlooked over the last 50 years, as reflected by the limited availability of clinical intervention studies, the absence of evidence-based international management guidelines and substantial gaps in understanding its long-term outcomes and underlying pathophysiological mechanisms (2).

In 2020, the global number TB survivors was estimated at approximately 155 million (3). TB was responsible for around 122 million disability-adjusted life years, of which nearly 58 million were associated to post-TB morbidity and illness instead of active infection, highlighting its considerable clinical and socioeconomic burden in low-and middle-income countries (4). TB survivors may lose up to 48% in quality-adjusted life expectancy due to post TB sequelae. Differences in the terminology and definition of post-tuberculosis lung disease across studies have limited comparability and integration of the available evidence (5). Terms such as *post-tubercular sequelae, post-pulmonary tuberculosis, chronic lung impairment following pulmonary tuberculosis (CLIPTB), post-tuberculosis bronchiectasis*, and *post-tuberculosis lung disorder* have been used to describe overlapping but not always identical clinical entities. This inconsistent nomenclature has likely contributed to fragmented evidence, complicated literature retrieval and evidence synthesis, and hindered the development of standardized clinical recommendations. Therefore, systematically documenting the terminology used across the literature is an important step toward improving consistency, facilitating future evidence synthesis, and supporting the development of consensus definitions for PTLD. To address this issue, the First International Symposium on Post-Tuberculosis Disease proposed defining PTLD as a chronic respiratory abnormality, symptomatic or asymptomatic, that is at least partially attributable to prior pulmonary tuberculosis. To address this issue, the first International symposium on post-TB disease utilized the definition of PTLD as a chronic respiratory abnormality, symptomatic or asymptomatic, that is at least partially attributable to prior pulmonary tuberculosis (6). The risk of death among TB survivors is three to six times greater, with the highest mortality seen in the first year after completing treatment (7). The diagnosis of PTLD is challenged by its diverse clinical presentation and wide spectrum of disease course, necessitating a tailored individual approach. Spirometry commonly demonstrates obstructive, restrictive or mixed ventilatory patterns. Pulmonary function abnormalities are observed in approximately 50–60% of TB survivors, while severe pulmonary impairment is reported in 10–15% of patients (7). Chest image including radiography and high-resolution computed tomography identifies structural lung damage.

Patients with PTLD may have a multi-factorial degree of functional and structural sequelae of the lung, including airways (obstructive lung disease and bronchiectasis), parenchyma (fibrotic changes, parenchymal destruction, and aspergillus lung disease), chronic pleural disease and pulmonary vascular complications (pulmonary hypertension) (8). These patients may also experience reduced exercise tolerance, diminished quality of life, and respiratory symptoms such as breathlessness and cough (9). There is growing evidence that up to 50% of TB patients continue to have health problems even after completing a full course of anti-tuberculosis treatment (10–12). Consequently, PTLD poses a significant burden on the global community, thus stressing the importance of prevention, management, and rehabilitation (9).

PR improves multiple outcomes in respiratory disease patients, including exercise capacity, reduced depression and anxiety levels, improved health-related quality of life (HRQOL), reduced fatigue, and increased respiratory muscle strength (13). It is well-known that PR is effective in patients with CRD such as chronic obstructive pulmonary disease (COPD), interstitial lung disease (ILD), bronchiectasis, idiopathic pulmonary fibrosis (IPF), and asthma (14). Give the substantial burden of post-tuberculosis lung disease and the established role of pulmonary rehabilitation in chronic respiratory diseases, this systematic review synthesized evidence from the randomized and observational interventional studies to determine the effectiveness of pulmonary rehabilitation, compared with usual care or no pulmonary rehabilitation, in improving exercise capacity, pulmonary function, health-related quality of life and respiratory symptoms in adults with post-tuberculosis lung disease.

Our objectives were threefold: (1) to systematically map the characteristics of PR interventions implemented for PTLD, including program components, duration, and delivery settings; (2) to synthesize available evidence on key outcomes, exercise capacity, quality of life, pulmonary function, and symptom burden; and (3) to identify methodological gaps and research priorities to inform future clinical trials and practice guidelines. To our knowledge, this is the first systematic review to comprehensively examine PR interventions specifically for PTLD, distinguishing this population from broader chronic respiratory disease cohorts. To address these objectives systematically, we conducted a systematic review following established PRISMA 2020 guidelines, as detailed in the following Methods section.

## Methods

### Search strategy and selection criteria

We conducted a comprehensive literature search following the most recent guidelines of the Preferred Reporting Items for Systematic Review and Meta-Analysis (PRISMA 2020) (15). The review protocol was not registered. Nevertheless, the review was conducted according to the predefined eligibility criteria and a prespecified search strategy established before study selection to enhance methodological transparency and minimize selection bias. However, the review was guided by predefined eligibility criteria and a prespecified search strategy established before study selection to minimize the risk of *post-hoc* decision-making and enhance methodological transparency. In 2026 we searched various databases for peer-reviewed articles in PubMed (Medline), Scopus, ProQuest, and EBSCO. The review question and study eligibility criteria were developed according to the Population, Intervention, comparator, Outcome and Study design (PICOS) framework. An information specialist iteratively designed and executed the search strategy using search terms “post-tuberculosis,” “tuberculosis sequelae,” “post-TB sequelae,” “chronic TB,” and also “pulmonary rehabilitation,” “respiratory rehabilitation,” “lung rehabilitation,” “breathing exercise” and “physical exercise”; excluding records that do not explicitly mention PTLD. The search was limited to most recent articles published from January 2000 to May 2026. Controlled vocabulary and free-text terms were used as appropriate and adapted for each database. References were gathered and arranged using the Endnote X8 version. Two reviewers independently screened titles and abstracts using the Endnote software. We included studies that evaluated PR or exercise-based interventions in adults with PTLD, with the aim describing intervention characteristics (type, duration, setting) and synthesizing outcomes related to exercise capacity, quality of life, pulmonary function, and symptoms. Conversely, we excluded reviews, protocols, guidelines, and literature primarily reporting on a specific lung condition and evaluating individuals with a history of TB only as a subgroup without reporting the main study outcomes. Studies describing the implementation of guidelines or protocols, and reviews not specifically focused on post-pulmonary tuberculosis, were also excluded. Only published studies employing PR as an intervention for patients diagnosed with PTLD were analyzed.

### Data items and charting process

Relevant literature was identified through a predefined search strategy, and data were extracted using standardized charting form, capturing the predefined study characteristics, such as author, title, keywords, publication year, type of PTLD addressed, and primary outcomes. All intervention types were deemed eligible, provided they incorporated PR aimed at improving quality of life (QOL), exercise tolerance, psychological aspects, breathlessness, and pulmonary functions in PTLD patients. The delivery mode for PR could include institutional settings, center-based, home-based, telerehabilitation, or community settings, and could have been implemented for any duration. Outcomes related to QOL, exercise capacity/functional capacity, breathlessness, lung function, respiratory muscle strength, and psychological aspects were included in the systematic review.

**Figure 1:**
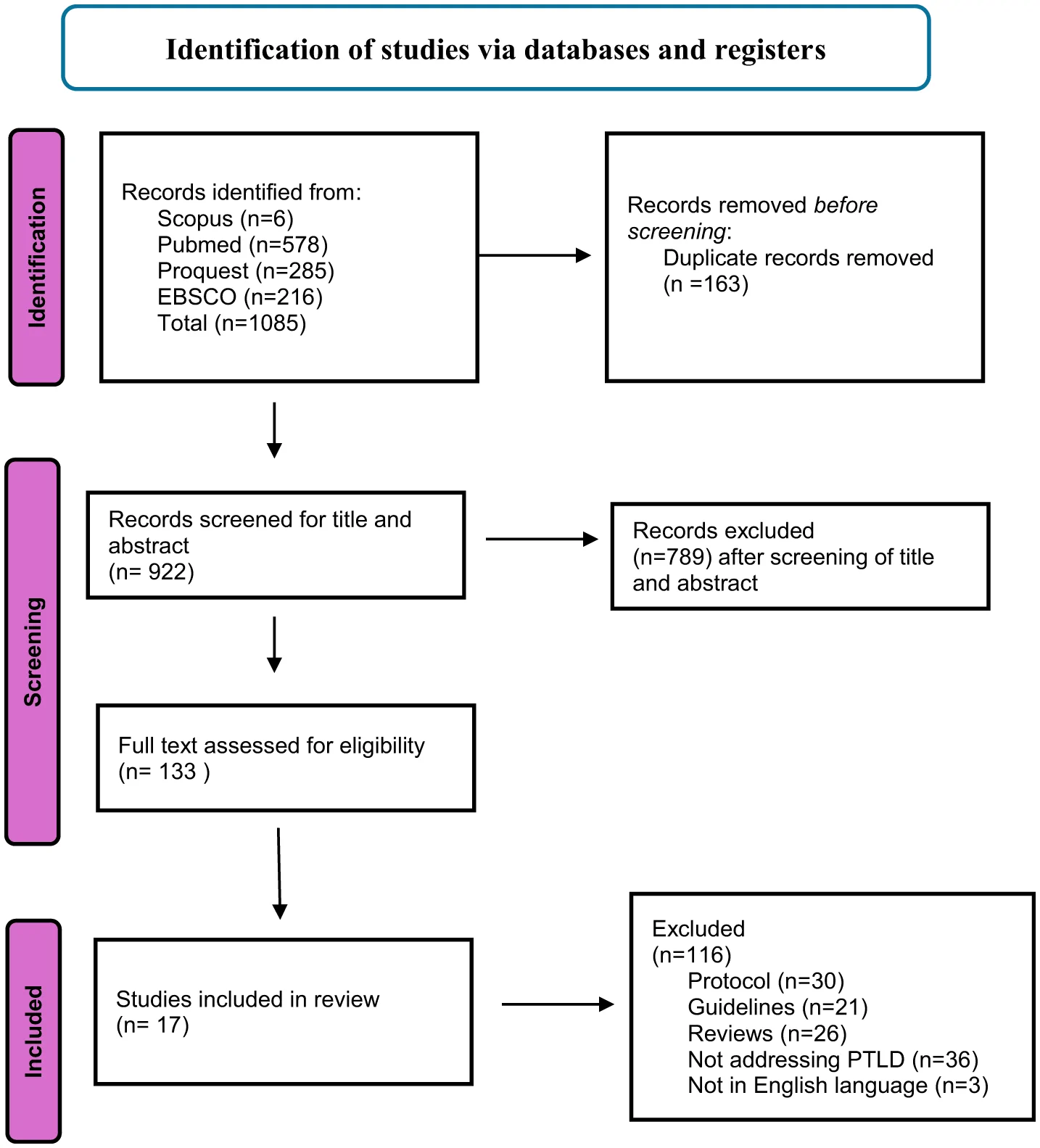
PRISMA flowchart.

The initial search identified a total of 1085 records, distributed as follows: Scopus = 6, PubMed (Medline) = 578, EBSCO = 216, and ProQuest = 285. After the initial screening, we removed 163 duplicates. The titles and abstracts of the remaining 922 articles were independently screened by two reviewers. Discrepancies were resolved through consensus, with arbitration by a third reviewer when required. Subsequently, a total of 789 articles were excluded. The remaining 133 studies were read entirely, and an additional 116 studies were eliminated for not meeting the inclusion criteria. Ultimately, 17 studies were included in our systematic review. Figure 1 illustrates the PRISMA flowchart.

Two reviewers independently extracted data, and any discrepancies were resolved through discussion or consultation with a third member of the team. The reviewers performed an initial calibration exercise to ensure consistency of the data extraction process.

### Outcome synthesis and evidence classification

**Figure 2:**
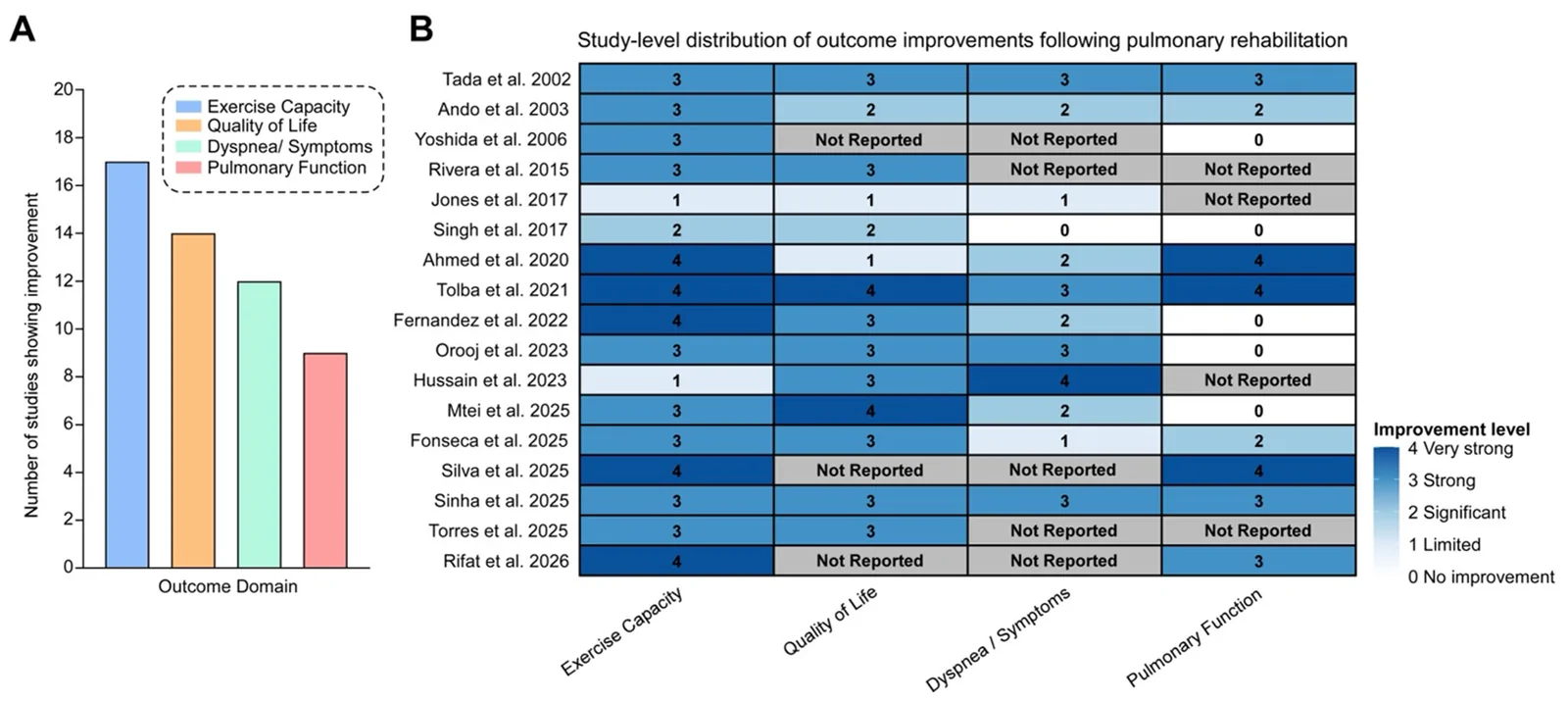
Study-level distribution of outcome improvements. **(A)** Number of studies reporting improvement across outcome domains. **(B)** Study-level distribution of improvement across outcome domains.

To facilitate visual synthesis of the heterogeneous outcome measures reported across studies, a descriptive classification was applied to each study and outcome domain (Figure 2). Because the included studies used different outcome measures to assess similar clinical domains (e.g., exercise capacity assessed by the 6 min walk distance, Incremental Shuttle Walk Test, or peak oxygen uptake), outcomes were classified according to the strength and consistency of the evidence for improvement reported within each study. The classification was based on three predefined criteria: (1) the number of outcome measures or subdomains demonstrating improvement within each domain; (2) the statistical support for the reported findings (e.g., statistically significant within-group or between-group differences); and (3) the consistency of improvement across multiple assessments or follow-up evaluations. Scores ranged from 0 to 4, where **0** indicated that the outcome was assessed but no directional or statistically significant improvement was reported; **1** indicated descriptive or within-group improvement without formal statistical support or without a significant comparison with a control group; **2** indicated statistically supported improvement limited to a single measure or accompanied by mixed findings within the domain; **3** indicated consistent improvement supported by multiple measures or subdomains, a significant comparison with a control group, or maintenance of benefit at follow-up; and **4** indicated robust and consistent improvement across multiple measures or subdomains with additional support from controlled comparisons and/or a large reported effect. Outcomes that were not assessed were categorized separately as “Not reported.”

### Risk of bias assessment

Risk of bias was independently assessed by two reviewers according to study design. The three randomized controlled trials were evaluated using the Cochrane Risk of Bias 2 (RoB 2) tool, whereas the 14 non-randomized intervention studies were assessed using the Risk of Bias in Non-randomized Studies of Interventions (ROBINS-1) tool. Any disagreements were resolved through discussion until consensus was reached.

## Results

**Figure 3:**
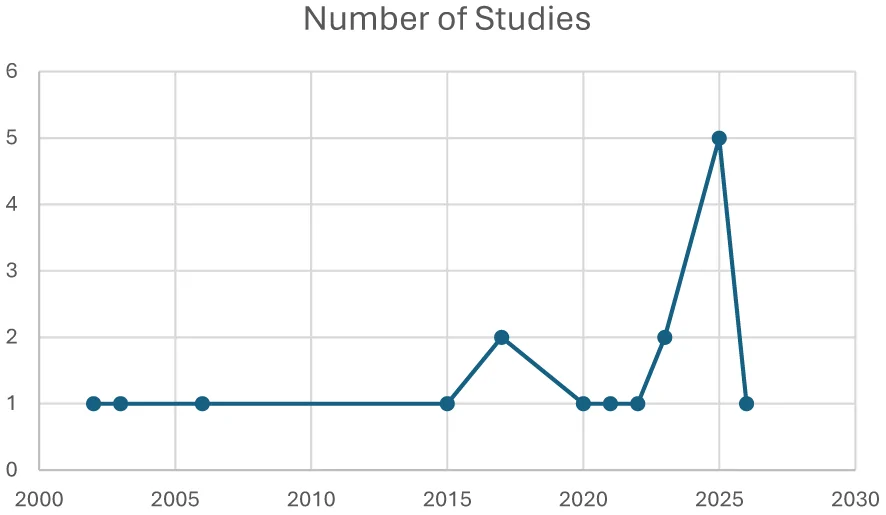
Publication trend of studies on pulmonary rehabilitation in post-tuberculosis lung disease (2002–2006).

We included 17 studies in this review. Table 1 presents the characteristics of these studies. The study designs were heterogeneous and included RCTs, prospective population-based studies, quasi-experimental studies, prospective cohort studies, prospective interventional studies, observational studies, retrospective analyses, longitudinal studies, and pre-post interventional studies without control groups. The studies were conducted across multiple countries, including India, Bangladesh, Brazil, Tanzania, Egypt, Uganda, Japan, Colombia, and the Philippines. Most of the studies were hospital- or institution-based, while several recent studies explored home-based, community-based, and tele-rehabilitation approaches for PR in patients with PTLD. The majority of the studies utilized PR as the primary intervention, incorporating components such as aerobic training, breathing exercises, resistance training, stretching, inspiratory muscle training, airway clearance techniques, and exercise re-training. The duration of PR interventions varied from 2 weeks to 24 weeks. Across the studies, PR demonstrated improvements in exercise capacity, dyspnea, pulmonary function, quality of life, mental health, and functional status among patients with PTLD. The included studies demonstrated a noticeable increase in research activity over time (Figure 3). Between 2002 and 2015, only four studies evaluating pulmonary rehabilitation in post-tuberculosis lung disease were identified. In contrast, thirteen studies were published between 2017 and 2026, with the highest number published during 2023–2026.

**Table 1:** Characteristics of the included studies.

S.No	Study	Full article/Abstract	Design	Country	Setting	Patient Population	Total Sample Size (*n*)	Participants completed the study (*n*)	Duration	Intervention	Outcome
1	Rifat et al. (24)	Full article	Prospective population based study	Bangladesh	Hospital	Post-TB lung disease	*n* = 202	*n* = 202	6 weeks	Warm-up, aerobic exercises, resistance exercises and cool-down	PR helps to improve lung function and exercise capacity in patients with PTLD.
2	Sinha et al. (30)	Full article	Multi-centric pre-and post-interventional study	India	Tele-rehabilitation/home	Post tuberculosis lung disease	*n* = 260	*n* = 246	12 weeks	Stretching, endurance and strength training, breathing exercises, respiratory muscle exercises with incentive spirometer and airway clearance techniques with postural drainage.	PR is beneficial in PTLD patients leading to improvement in exercise capacity, symptom management as well the overall quality of life.
3	Fonseca et al. (31)	Full article	Quasi-experimental study	Brazil	Home-Based	Post tuberculosis lung disease	*n* = 26	*n* = 23	12 weeks	Breathing exercises, aerobic training, muscle strengthening, flexibility exercise and postural control training.	Home-based PR improves exercise tolerance with PTLD patients.
4	Mtei et al. (25)	Full article	Prospective, non-controlled interventional study	Tanzania	Community-Based	Post-TB lung disease	*n* = 121	*n* = 121	24 weeks	Aerobic and resistance training,	Community based PR improves symptomatic individual's quality of life, physical capacity and mental health.
5	Torres et al. (26)	Full article	Randomized controlled trial	Brazil	Tele-rehabilitation	Post-TB lung disease	*n* = 30	*n* = 30	8 weeks	Aerobic training, breathing exercises, strength training and stretching exercises.	Telerehabilitation is effective in enhancing physical function and quality of life in patients with PTLD.
6	Silva et al. (27)	Full article	Prospective multicentred interventional	Brazil, Italy, France	Outpatient reference centers	PTLD	*n* = 181	*n* = 181	5 weeks	Aerobic training, Bronchial hygiene, Maintenance physical exercises and home oxygen therapy.	Improved benefits from PR program with PTLD patients including improvements in lung function and exercise capacity.
7	Orooj et al. (16)	Full article	Randomized control trial	India	Institutional	Post pulmonary tuberculosis	*n* = 90	*n* = 90	16 Sessions/4 weeks (4 sessions per week)	Breathing exercises, supervised endurance training (walking), supervised resistance training, stretching of upper and lower extremity muscle.	Short term PR in post PTB showed improvement in exercise capacity, quality of life and dyspnea.
8	Hussain et al. (20)	Full article	Hospital based longitudinal study	India	Hospital	Post tuberculosis sequelae	*n* = 41	*n* = 26	6 weeks	Treadmill, stationary cycling, strengthening exercises for quadriceps and upper limbs and breathing exercises.	PR has improved dyspnea, functional capacity, quality of life and mental health impairment in Post tuberculosis sequelae patients.
9	Fernandez et al. (32)	Full article	Prospective study	Philippines	Hospital	Post tuberculosis bronchiectasis	*n* = 14	*n* = 14	4 weeks	Breathing re-training, dyspnea-relieving techniques, upper and lower limb resistance training.	PR has led to a significant improvement in exercise capacity dn health-related quality of life among post-tuberculosis bronchiectasis.
10	Tolba et al. (29)	Full article	Prospective comparative interventional study	Egypt	Hospital	Treated pulmonary tuberculosis	*n* = 60	*n* = 60	60 min/sessions for 3 days, 12 weeks	Treadmill, upper and lower limb exercise training, inspiratory muscle training.	Significant improvement in terms of exercise capacity, lung function and health related quality of life in treated PTB patients.
11	Ahmed et al. (17)	Full article	Single blind randomized controlled trial	India	Hospital	Post tubercular sequelae	*n* = 62	*n* = 62	12 weeks/1 h/day for 5 days in a week	Breathing exercises, Walking and Inspiratory muscle training.	Beneficial in reducing residual pulmonary impairment.
12	Singh et al. (21)	Full article	Prospective cohort study	India	Institutional	Chronic lung impairment from pulmonary tuberculosis (CLIPTB)	*n* = 45	*n* = 29	3 sessions/week, 8 weeks, 90 min/day	Leg ergometry and treadmill walking.	PR is effective in post tuberculosis patients quality of life and functional status.
13	Jones et al. (22)	Full article	Pre and post interventional cohort study	Uganda	Hospital	Post tuberculosis lung disease	*n* = 34	*n* = 29	2 sessions/week for 6 weeks, 2 h/day	Walking, sit to stand, step-ups, biceps curls, upright rowing with weights.	PR with PTLD patients was feasible and improves quality of life, exercise capacity and respiratory outcomes.
14	Rivera et al. (28)	Abstract	Pre and post design without control group	Colombia	Institutional	Sequelae of pulmonary TB	*n* = 8	*n* = 8	3 days/week for 8 weeks	Treadmill, warm-up and stretching exercises.	PR resulted in significant improvement in both aerobic and exercise capacity.
15	Yoshida et al. (18)	Full article	Observational study	Japan	Hospital	Pulmonary tuberculosis sequelae	*n* = 14	*n* = 10	2 weeks, 2 sets per/day	Pursed lip, diaphragmatic breathing exercise and walking	Exercise training is safe and effective for the improvement of exercise performance with pulmonary tuberculosis sequelae patients.
16	Tada et al. (19)	Full article	Retrospective analysis	Japan	Hospital	Pulmonary tuberculosis sequelae	*n* = 37	*n* = 37	3.9 weeks	Relaxation, breathing retraining, exercises training, respiratory muscle training and instruction.	PR improves pulmonary function, exercise tolerance, symptoms and quality of life in patients with pulmonary tuberculosis sequelae.
17	Ando et al. (23)	Full article	Prospective nonrandomized trial	Japan	Hospital	Post tuberculosis lung disorder	*n* = 32	*n* = 32	9 weeks	Relaxation, pursed-lip breathing and slow-deep breathing exercise, low-intensity strength training of the upper and lower extremities.	PR is beneficial in post-TBC lung disorder.

Studies using varying terminology to describe PTLD. Orooj et al. (16) described it as post-pulmonary tuberculosis, whereas Ahmed et al. (17), Yoshida et al. (18), Tada et al. (19), and Hussain et al. (20) referred to it as post-tubercular sequelae or pulmonary tuberculosis, whereas Sing et al. (21) referred to it as chronic lung impairment from pulmonary tuberculosis (CLIPTB). Jones et al. (22), Ando et al. (23), Rifa et al. (24), Mtei et al. (25), Torres et al. (26), and Silva et al. (27) specifically used the term PTLD. Other related terminologies include sequelae of pulmonary TB (28), treated pulmonary tuberculosis (29), post-tuberculosis bronchiectasis (30), post-tuberculosis lung disorder (31), and sequelae of pulmonary tuberculosis (32). Supplementary Table S1 presents the terminology used across studies.

### Risk of bias assessment

**Figure 4:**
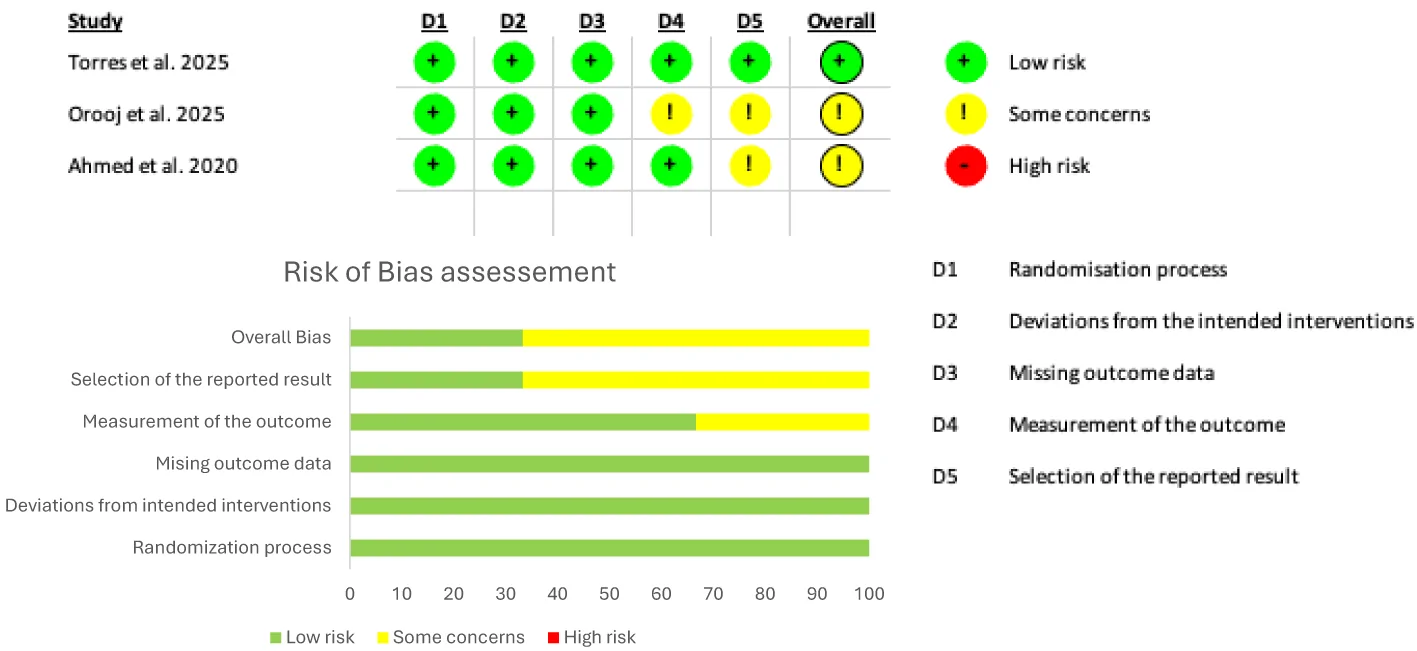
RoB 2 risk of bias assessment of included randomized controlled trials.

The risk-of-bias assessment identified varying methodological quality among the included studies. Of the three randomized controlled trials, one study was judged to have a low overall risk of bias, whereas two studies were judged to have some concerns, primarily because of potential bias in the measurement of outcomes and selection of the reported results. Among the 14 non-randomized intervention studies to have a moderate overall risk of bias. The most common source of bias among the non-randomized studies was bias due to confounding, reflecting the predominance of uncontrolled or non-randomized study designs. In contrast, bias in the classification of interventions was consistently rated as low across all non-randomized studies. Detailed assessments are presented in Figure 4 and Table 2.

**Table 2:** ROBINS-1 risk of bias assessment of the included non-randomized studies.

Study	Bias due to confounding	Bias in selection of participants	Bias in classification of Interventions	Bias due to deviation from intended interventions	Bias due to missing data	Bias in measurement of outcomes	Bias in selection of the reported result	Overall ROBINS-1
Tada et al. (19)	Serious	Moderate	Low	Moderate	Moderate	Moderate	Moderate	Serious
Ando et al. (23)	Moderate	Low	Low	Low	Low	Moderate	low	Moderate
Yoshida et al. (18)	Serious	Serious	Low	Low	Low	Low	Moderate	Serious
Rivera et al. (28)	Serious	Serious	Low	Moderate	Low	Moderate	Moderate	Serious
Jones et al. (22)	Serious	Moderate	Low	Moderate	Moderate	Moderate	Low	Serious
Sing et al. (21)	Serious	Moderate	Low	Moderate	Serious	Moderate	Moderate	Serious
Tolba et al. (29)	Moderate	Moderate	Low	Low	Low	Moderate	Moderate	Moderate
Fernandez et al. (32)	Serious	Moderate	Low	Moderate	Low	Moderate	Moderate	Serious
Hussain et al. (20)	Serious	Moderate	Low	Moderate	Serious	Moderate	Moderate	Serious
Fonseca et al. (31)	Serious	Moderate	Low	Moderate	Low	Moderate	Low	Serious
Mtei et al. (25)	Serious	Moderate	Low	Low	Low	Moderate	Low	Serious
Silva et al. (27)	Moderate	Moderate	Low	Low	Low	Low	Low	Moderate
Sinha et al. (30)	Serious	Moderate	Low	Moderate	Low	Moderate	Low	Serious
Rifat et al. (24)	Serious	Moderate	Low	Moderate	Low	Low	Moderate	Serious

### Exercise capacity

Exercise capacity was the most consistently improved outcome across the included studies. Several outcome measures were used to assess changes in exercise capacity across the included studies. The Six-Minute Walk Distance (6MWD), Incremental Shuttle Walking Test (ISWT), and peak oxygen uptake (VO_2_peak) were commonly utilized. Of the 17 studies, 16 reported improvements in exercise capacity following pulmonary rehabilitation, while one study reported improvement that did not reach statistical significance. Improvements in 6 min walk distance generally ranged from approximately 36 to 189 m, with several studies exceeding the minimal clinically important difference for chronic respiratory diseases. These benefits were observed across randomized controlled trials, prospective cohorts, and pre-post intervention studies despite differences in rehabilitation protocols and program duration.

Ahmed et al. (17) found that 12 weeks of PR significantly improved 6MWD in the intervention group (+134 m), increasing from 440.65 ± 70.86 m at baseline to 574.6 m post-intervention, compared with the control group (+58.5 m), which improved from 462.03 ± 69.83 m to 520.48 m. Endpoint SDs were not reported for either group in the source study. Between-group analysis demonstrated a significant difference in 6MWD (mean difference 75.58 m; 95% CI: 49.57–101.60 m; *p* < 0.001). The authors reported a significant improvement in dyspnea scores; however, the reduction in St. George's Respiratory Questionnaire (SGRQ) scores did not differ significantly between the two groups by the end of the study. Singh et al. (21) similarly reported a significant increase in 6MWD, rising from 488 ± 110.1 m at baseline to 526 ± 81.2 m after 8 weeks of PR (*p* = 0.033). Tolba et al. (29) reported that after 12 weeks of PR, 6MWD increased from 411.1 ± 134.4 m to 600.3 ± 168.9 m in the intervention group, whereas the control group increased from 409.8 ± 143.6 m to 441.7 ± 151.2 m. A statistically significant post-treatment mean difference of 158.6 m was observed between the groups (*p* < 0.001) (29). Rivera et al. (28) reported that 6MWD increased by 63.6 m following an 8-week PR program (*p* = 0.014), whereas VO_2_ peak increased by 1.7 mL/kg/min (*p* = 0.039). Measures of variability were not reported. Yoshida et al. (18) found that 6MWD increased from 399 ± 62 m to 467 ± 65 m (*p* < 0.01), while VO_2_peak increased from 13.6 ± 2.8 to 14.8 ± 2.8 mL/kg/min (*p* < 0.01) after only 2 weeks of exercise training. Orooj et al. (16) reported a statistically significant improvement in 6MWD following a 4-week rehabilitation program. In the rehabilitation group, 6MWD increased from 286.85 ± 44.64 m at baseline to 484.15 ± 48.01 m post-intervention, whereas the control group showed only a modest increase from 313.0 ± 48.17 m to 321.32 ± 43.46 m (*p* < 0.001 for distance in meters). Similarly, the percentage predicted 6MWD improved from 64.2 ± 13.6% to 83.2 ± 11.4% in the rehabilitation group compared with minimal change in the control group (*p* = 0.014) (16). Similarly, Ando et al. (23) reported a significant increase in 6MWD of approximately 42 ± 60 m following a 9-week PR program (*p* < 0.01), alongside parallel improvements in activity levels and functional status. Furthermore, Tada et al. (19) observed an increase in 6MWD from 303 ± 64 m to 339 ± 61 m (*p* < 0.001), reinforcing consistent gains in exercise tolerance across studies. Jones et al. (22) reported improved exercise capacity in patients with PTLD, with ISWT distance increasing from 312.4 m (95% CI: 275.95–348.8) at baseline to 402.4 m (95% CI: 357.74–447.0) post-rehabilitation, with improvements maintained at the 6-week follow-up (402.0 m; 95% CI: 353.8–450.2). The mean improvement from baseline to the end of rehabilitation was 90 m (95% CI: 56.50–123.50), which remained sustained at 6 weeks (89.6 m; 95% CI: 49.32–129.9). This was a developmental study; outcomes were summarized descriptively without formal statistical significance testing, and changes were interpreted in relation to established minimal clinically important differences (22). Rifat et al. (24) demonstrated significant improvements in exercise capacity, with 6MWD increasing from 402.8 ± 109.1 m at baseline to 456.7 ± 108.6 m after rehabilitation (*p* < 0.0001).

Similarly, Torres et al. (26) reported notable improvement in exercise capacity following 8 weeks of PR. The intervention group demonstrated an increase in 6MWD from 501.5 ± 146.6 m to 571.2 ± 134.4 m, whereas only minimal improvement was observed in the control group (505.5 ± 97.1 m to 511.2 ± 96.9 m). Sinha et al. (30) also observed significant enhancement in functional capacity, with 6MWD increasing from 364.1 ± 146.1 m to 395.07 ± 157.7 m following PR (*p* < 0.001). Likewise, Mtei et al. (25) demonstrated significant improvement in exercise tolerance, with 6MWD increasing from 420 ± 87.3 m at baseline to 460 ± 61.96 m at week 24 (*p* < 0.001). Fernandez et al. (32) further reported marked improvement in 6MWD from 355.5 ± 94.9 m to 470.1 ± 76.9 m following rehabilitation (*p* < 0.001). Silva et al. (27) reported significant improvement in the intervention group, with 6MWD increasing from 367.5 ± 132.9 m at baseline to 425.9 ± 135.6 m following PR (*p* < 0.001). In contrast, the control group declined from 432.9 ± 99.7 m at baseline to 365.8 ± 104.2 m after 3 months (*p* < 0.001). Hussain et al. (20) reported an increase in 6MWD from 408.6 ± 108.3 m to 442.7 ± 107.5 m; however, this improvement was not statistically significant (*p* = 0.300).

### Pulmonary functions

Changes in pulmonary function measurements also varied across the included studies. Ahmed et al. (17) reported that in the intervention group, forced expiratory volume in 1st second (FEV1) increased significantly from 2.94 ± 0.67 L at baseline to 3.18 L (mean change: 0.239 L, *p* < 0.001), and the forced vital capacity (FVC) also increased significantly from 3.43 ± 0.74 L to 3.74 L (mean change: 0.31 L, *p* < 0.001). Post-intervention standard deviations were not reported. In contrast, the control group showed a smaller, yet significant increase in FEV1 from 2.85 ± 0.51 L to 2.89 L (*p* < 0.001), and a non-significant change in FVC from 3.52 ± 0.93 L to 3.68 L (*p* = 0.097). Between-group analyses showed the intervention was significantly superior for improving both FEV1 and FVC. Tolba et al. (29) also reported that in the rehabilitation group, FEV1 percent predicted increased from 68.43 ± 13.93 to 86.93 ± 7.62, FVC percent predicted increased from 85.99 ± 8.67 to 93.92 ± 4.82, and FEV1/FVC ratio increased from 79.5 ± 13.35 to 92.52 ± 6.04. In the control group, FEV1 percent predicted increased from 67.25 ± 14.8 to 69.86 ± 14.43, FVC percent predicted from 84.58 ± 10.01 to 85.71 ± 9.82, and FEV1/FVC ratio from 79.13 ± 12.43 to 81.2 ± 12.03. Post-treatment mean differences between the two groups were 8.2 for FVC% (*p* < 0.0001), 17.07 for FEV1% (*p* < 0.0001), and 11.32 for FEV1/FVC (*p* < 0.0001). Tada et al. (19) reported statistically significant improvements in both VC from 1.48 ± 0.47 L to 1.59 ± 0.51 L (*p* < 0.001) and FEV1 from 0.93 ± 0.33 L to 1.02 ± 0.37 L (*p* < 0.01). In contrast, Orooj et al. (16) found no statistically significant changes in pulmonary function measures after the 4-week rehabilitation program, despite significant improvements in functional capacity and quality of life. In the PR group, FEV1 changed from 1.29 ± 0.44 L to 1.44 ± 0.64 L (*p* = 0.142 vs. controls), FVC was unchanged at 1.64 ± 0.47 L pre- and post-intervention (*p* = 0.115 vs. controls), and the FEV1/FVC ratio remained exactly 100.37 ± 16.0. Singh et al. (21) similarly reported that pulmonary function tests showed a trend toward improvement, but the changes did not reach statistical significance. Following 8 weeks of PR, FEV1 changed from 1.02 ± 0.32 L to 1.07 ± 0.33 L (*p* = 0.62), FVC from 1.76 ± 0.54 L to 1.81 ± 0.56 L (*p* = 0.74), and FEV1/FVC ratio from 60.98 ± 16.37 to 60.55 ± 15.26 (*p* = 0.92). Yoshida et al. (18) also reported no significant changes in pulmonary function tests or arterial blood gas parameters following 2 weeks of walking training, with VC changing from 1.41 ± 0.37 L to 1.38 ± 0.29 L and FEV1 changing from 0.95 ± 0.45 L to 0.95 ± 0.38 L. Also, Ando et al. (23) found no significant changes in FEV1 after a 9-week rehabilitation program (mean increase of 28 mL [SD 87]; 95% CI: −4 to 59 mL), but a modest significant increase in vital capacity was observed in post-tuberculosis patients (mean increase of 72 mL [SD 105]; 95% CI: 34 to 109 mL, *p* < 0.01).

More recent studies have also reported mixed findings regarding pulmonary function. Rifat et al. (24) reported statistically significant improvements in spirometric parameters with FEV1 percentage predicted increasing from 44.6 ± 14.1 to 47.9 ± 14.2 (*p* < 0.0001) and FVC percentage predicted increasing from 55.6 ± 13.3 to 58.3 ± 13.4 (*p* < 0.0001), whereas no significant change was observed in the FEV1/FVC ratio, which changed from 64.4 ± 13.3 to 65.3 ± 12.8 (*p* = 0.468). Similarly, Sinha et al. (30) demonstrated significant improvements in median FEV1 and FVC values following a PR program. FEV1 improved from 1.16 L (range: 0–3.81) to 1.38 L (range: 0.44–4.03) (*p* < 0.001), and FVC improved from 2.04 L (range: 0–4.3) to 2.38 L (range: 1–49) (*p* < 0.001), although the FEV1/FVC ratio did not significantly change (66.3 [range: 0–100] to 79 [range: 0.24–100], *p* = 0.590). Silva et al. (27) demonstrated significant improvements in aggregate pulmonary function parameters. In the rehabilitation group, post-bronchodilator FEV1 increased from 1.33 ± 0.85 L to 1.56 ± 0.93 L, FVC increased from 2.37 ± 0.92 L to 2.61 ± 1.03 L, and the FEV1/FVC ratio increased from 59.2 ± 23.5% to 62.3 ± 20.9%. In contrast, the non-rehabilitation group showed a decline in FEV1 (1.66 ± 0.84 L to 1.39 ± 0.76 L), FVC (2.52 ± 1.01 L to 2.26 ± 0.93 L), and FEV1/FVC ratio (65.7 ± 17.7% to 60.5 ± 19.3%). Significant between-group differences favored the rehabilitation group (*p* < 0.0001). In contrast, Fonseca et al. (31) observed no statistically significant changes in spirometric data following home-based PR, with FVC percentage predicted changing from 78.8 ± 20.7 to 79.2 ± 19.4 (*p* = 0.74), FEV1 percentage predicted from 75.5 ± 25.7 to 76.2 ± 25.1 (*p* = 0.57), and median FEV1/FVC ratio changing from 82 (IQR: 68–86) to 83 (IQR: 68–87) (*p* = 0.76). Similarly, Mtei et al. (25) found no significant improvements in median pulmonary function measurements over 24 weeks, including FEV1% predicted (67.00 [IQR: 49–87.25] to 69.50 [IQR: 51.25–91.75]; *p* = 0.288), FVC% predicted (74.00 [IQR: 59.75–93.25] to 77.00 [IQR: 62.25–98.75]; *p* = 0.189), and FEV1/FVC ratio (69.60 [IQR: 68.20–70.70] to 69.40 [IQR: 68.20–70.70]; *p* = 0.400). Fernandez et al. (32) also reported non-significant changes in the pulmonary function parameters FEV1 (54.4 ± 24.0% to 51.6 ± 21.9%, *p* = 0.14), FVC (63.2 ± 19.7% to 62.5 ± 18.4%, *p* = 0.640), and FEV1/FVC (69.0 ± 15.2% to 67.7 ± 15.3%, *p* = 0.500).

### Quality of life

Health-related quality of life improved consistently across the included studies despite the use of different assessment instruments. Twelve of the 13 studies evaluating quality of life reported significant improvements following pulmonary rehabilitation, while one study found no significant between-group difference. Positive effects were observed across multiple domains, including symptoms, physical functioning, activity limitation, and overall health status, suggesting that the benefits of pulmonary rehabilitation extend beyond improvements in exercise performance alone.

Tolba et al. (29) reported between-group differences across all domains of the SGRQ. In the rehabilitation group, the SGRQ symptom score decreased from 27.8 ± 10.67 to 16.9 ± 7.96, activity score from 28.6 ± 9.05 to 15.7 ± 5.59, impact score from 24.63 ± 10.77 to 14.3 ± 7.75, and total score from 27.23 ± 11.87 to 15.66 ± 8.18. In the control group, scores decreased slightly from 28.93 ± 11.92 to 27.13 ± 11.59 for SGRQ symptoms, from 29.33 ± 11.13 to 27.6 ± 10.93 for activity, from 25.73 ± 11.41 to 24.46 ± 11.44 for impact, and from 28.13 ± 13.12 to 26.93 ± 13.12 for total score, with all post-treatment between-group differences reaching statistical significance at *p* < 0.0001. Rivera et al. (28) reported a 13-point improvement in the SGRQ score (*p* = 0.039) and a 6.98-point improvement in the physical domain of the SF-36 (*p* = 0.039); however, measures of variability were not reported in the abstract. Singh et al. (21) reported that the CRQ score increased from 17.21 ± 2.86 to 18.96 ± 2.91 following 8 weeks of PR, with *p* = 0.025. Jones et al. (22) reported that the Clinical COPD Questionnaire (CCQ) score decreased from 1.88 (95% CI: 1.59–2.17) at baseline to 0.93 (95% CI: 0.76–1.10) at the end of the PR program and further to 0.79 (95% CI: 0.63–0.96) at 6-week follow-up, indicating sustained improvement in health status after program completion; however, no formal statistical significance testing was performed. Orooj et al. (16) also reported significant improvement across all domains of the SF-36 questionnaire at the completion of the PR program (*p* < 0.05). For example, Physical Functioning improved from 42.4 ± 7.3 to 88.9 ± 6.3, while Mental Health improved from 65.1 ± 8.3 to 88.4 ± 5.1. Ando et al. (23) demonstrated improvements in activities of daily living (ADL) scores following a PR program (improving from a mean of 5.5 to 5.8 out of 6), reflecting enhanced functional independence. Similarly, Tada et al. (19) reported a significant increase in QOL scores from 39.0 ± 8.1 to 44.2 ± 8.8 (*p* < 0.01), further supporting the beneficial impact of PR on patient-reported outcomes. In contrast, Ahmed et al. (17) reported that the SGRQ score in the intervention group decreased from 24.53 ± 11.81 at baseline to 11.61 after 12 weeks, while the control group decreased from 24.69 ± 10.71 to 11.68; post-intervention standard deviations were not reported and the between-group difference was not statistically significant, *p* = 0.231.

More recent studies also demonstrated consistent improvements in health-related quality of life following PR. Torres et al. (26) reported significant reductions in SGRQ scores in the intervention group from 28.8 ± 21.6 to 17.6 ± 13.9 (*p* < 0.01), whereas no significant changes were observed in the control group. Significant improvements were also observed in the SF-36 physical function and mental health domains in the intervention group. Sinha et al. (30) similarly demonstrated marked reductions in median SGRQ symptom (58.6 [range: 0–389.8] to 30.95 [range: 0–112.8]), impact (52.9 [range: 0–457] to 31.1 [range: 0–456]), and activity scores (59.5 [range: 0–522.8] to 34.66 [range: 0–701]) following rehabilitation (*p* < 0.001 for all). Fonseca et al. (31) observed significant improvements in median SGRQ activity (60.3 [IQR: 20–80] to 35.5 [IQR: 0–60]; *p* = 0.012), impact (39.5 [IQR: 16–62] to 18.5 [IQR: 12–45]; *p* < 0.01), and total scores (37.5 [IQR: 18–68] to 22.4 [IQR: 9–51]; *p* < 0.001) following home-based PR; however, symptom scores did not significantly improve (37.3 [IQR: 20–59] to 17.3 [IQR: 12–48]; *p* = 0.14). Mtei et al. (25) reported substantial reductions across all SGRQ domains, including symptom (45.68 ± 16.56 to 21.10 ± 13.31), activity (46.98 ± 22.70 to 17.44 ± 13.47), impact (22.99 ± 14.99 to 6.06 ± 5.63), and total scores (34.63 ± 17.03 to 12.99 ± 9.67) (*p* < 0.001 for all). Fernandez et al. (32) also reported significant improvements, with the SGRQ total score improving from 39.0 ± 18.8 to 25.0 ± 12.6 (*p* < 0.01).

### Symptoms related outcomes

Symptom-related outcomes also generally favored pulmonary rehabilitation. Six of the seven studies assessing symptoms reported significant improvements in dyspnea, cough, chest pain, fatigue, or functional symptoms, whereas one study found no significant change in dyspnea. Although symptom assessment varied across studies, the overall findings consistently suggest that pulmonary rehabilitation alleviates symptom burden in individuals with post-tuberculosis lung disease.

Jones et al. (22) reported that the proportion of participants with chest pain fell from 13 out of 29 participants (44.8%) at baseline to 9 out of 29 participants (31%) at the end of the PR program and to 7 out of 29 (24.1%) at 6 weeks after the end of PR, while hemoptysis declined from 5 out of 29 participants (17.2%) at baseline to 2 out of 29 participants (6.9%) after the PR program and remained at 2 out of 29 participants (6.9%) at 6 weeks after the end of PR. The same study also reported improvements in sit-to-stand time, from 10.45 (95% CI: 9.29–11.61) seconds at baseline to 7.90 (95% CI: 7.01–8.78) seconds post-PR program, and 7.03 (95% CI: 6.53–7.54) seconds at follow-up, along with reductions in post-exercise Borg scores from 6.48 (95% CI: 5.68–7.28) to 4.36 (95% CI: 3.55–5.17) and 4.52 (95% CI: 3.65–5.39) 6 weeks after the end of PR. Orooj et al. (16) further reported statistically significant improvement in dyspnea using modified Medical Research Council (mMRC) scores. In the rehabilitation group, mMRC scores decreased from 3.38 ± 0.49 to 0.02 ± 0.12, whereas the control group changed from 3.60 ± 0.49 to 0.50 ± 0.65, yielding a significant between-group difference (*p* < 0.01).

Consistent with these findings, Ando et al. (23) reported significant improvements in both the Medical Research Council (MRC) dyspnea scale grade (improving from a mean of 3.4 to 3.1) and the Transition Dyspnea Index (TDI) (median +1, range 0–5) following a PR program. Tada et al. (19) likewise reported improvement in dyspnea scores from 18.4 ± 5.7 to 20.9 ± 5.5 (*p* < 0.05), alongside enhanced activity levels. Hussain et al. (20) reported significant reductions in mMRC dyspnea scores from 1.5 ± 0.6 to 0.7 ± 0.5 (*p* < 0.001). Sinha et al. (30) showed significant improvements in multiple clinical symptoms following PR. After 12 weeks of PR, cough prevalence reduced from 215/260 participants (83%) to 111/260 participants (43%) (*p* < 0.0001) and chest pain decreased from 59/260 participants (22%) to 27/260 participants (10%) (*p* < 0.001). Additionally, dyspnea severity improved significantly, with the number of participants in mMRC grade 3 decreasing from 33 (13%) pre-intervention to 16 (6%) post-intervention, while participants in mMRC grade 0 increased from 102 (39%) to 118 (46%) (*p* < 0.001).

In summary, despite variability in intervention types and outcome measures, most studies consistently demonstrate improvements in exercise capacity and health-related quality of life following PR. Functional outcomes demonstrated the most consistent and clinically meaningful improvements, whereas pulmonary function results were more variable across the 13 studies. Importantly, improvements in symptoms, such as dyspnea, chest pain, and fatigue, were also observed in studies where these outcomes were assessed. Figure 2A illustrates the number of studies reporting improvements across the outcome measures. Overall, every included study demonstrated improvement in at least one key outcome domain, reinforcing the potential value of PR as an effective intervention for individuals with PTLD. Figure 2B shows the magnitude of the improvement on each domain across the nine studies.

## Discussion

PR interventions consistently improved functional capacity and quality of life in PTLD patients across diverse settings, despite methodological heterogeneity and predominantly non-randomized designs. These findings address our three objectives: mapping intervention characteristics, synthesizing outcome evidence, and identifying research gaps. These results suggest that PR addresses a critical gap in post-TB care by targeting physical deconditioning and symptom burden that persist after microbiological cure. Below, we interpret these findings in relation to each outcome domain, discuss clinical implications, and highlight priorities for future research. Functional outcomes, especially exercise capacity showed the most consistent improvements following PR across the included studies. Several studies reported improvements following in 6 min walk distance that exceeded the commonly accepted minimal clinically important difference (MCID) of approximately 25–35 m for chronic respiratory diseases, suggesting that these benefits were clinically meaningful (33). These findings suggest that PR addresses a key gap in post-treatment care by targeting physical deconditioning and reduced endurance, which are not resolved through pharmacological treatment alone. Importantly, improvements in exercise performance were observed across different program durations and settings, indicating that even relatively short- or resource-limited interventions may provide measurable benefits. A recent systematic review by Safareena et al. (34) similarly evaluated the effects of pulmonary rehabilitation in individuals with post-tuberculosis lung disease and reported improvements in exercise capacity, functional status, and health-related quality of life, while noting that improvements in pulmonary function were less consistent across studies. Our findings are consistent with these observations and additionally provide a detailed synthesis of pulmonary rehabilitation program characteristics, including intervention components, duration, frequency, and delivery settings.

The predominance of non-randomized studies with serious risk of bias indicates that the current evidence base should be interpreted cautiously. The observed improvements in exercise capacity, quality of life and symptom burden are encouraging; however, the absence of randomization and concurrent control groups in many studies increases the possibility that factors other than pulmonary rehabilitation to the reported benefits. Nevertheless, the consistency of positive findings across randomized and non-randomized studies, together with the reproducibility of improvements across different countries and rehabilitation settings, supports the potential effectiveness of pulmonary rehabilitation in patients with post-tuberculosis lung disease.

The designs of these studies spanned across RCTs, prospective cohort studies, interventional cohort studies, and observational studies. The interventions in these studies involved various approaches, such as breathing exercises, endurance training, inspiratory muscle training, walking, and upper/lower limb exercises. These sessions were generally well tolerated and safe, with durations ranging from 2 weeks to several months. Across these studies, PR consistently showed positive effects on exercise capacity, QOL, and lung functions. For instance, several studies reported significant improvements in exercise capacity and QOL (16, 17, 29). While improvements in lung function were reported in some studies, the notable heterogeneity of the findings suggests that structural lung damage associated with PTLD may limit the extent of measurable recovery in spirometric parameters. This variability indicates that the benefits of PR may be driven more by peripheral adaptations, such as improved muscular efficiency and cardiovascular conditioning, rather than direct reversal of pulmonary impairment (35, 36). As a result, outcomes such as exercise capacity and quality of life may serve as more sensitive indicators of clinical improvement in this population.

A common theme in the current studies is the focus on individuals with post-pulmonary TB sequelae or chronic lung impairment resulting from pulmonary TB. These populations often face challenges related to reduced exercise tolerance, diminished lung function, and impaired QOL. The interventions discussed in these studies aimed to address these challenges and improve the overall wellbeing of these individuals. In addition, improvements in health-related quality of life and symptom burden across multiple studies highlight the multidimensional impact of PR. Reductions in dyspnea, fatigue, and activity limitation suggest that PR not only improves physical performance but also enhances patients' ability to engage in daily activities and social participation (37). These findings suggest that pulmonary rehabilitation primarily targets extrapulmonary factors, including skeletal muscle dysfunction and physical deconditioning, rather than reversing the underlying structural lung damage associated with PTLD. Many individuals with PTLD have irreversible anatomical abnormalities, including fibrosis, bronchiectasis, parenchymal destruction, and airway remodeling, which impose permanent mechanical limitations on lung function and may explain the relatively modest and heterogeneous improvements observed in spirometric parameters. In contrast, pulmonary rehabilitation improves peripheral conditioning through enhanced skeletal muscle efficiency, improved cardiovascular fitness, greater exercise tolerance, and reduced perception of dyspnea. Consequently, patients may experience clinically meaningful improvements in functional capacity and health-related quality of life despite limited changes in conventional pulmonary function measurements (38). Furthermore, pulmonary rehabilitation may promote important behavioral and psychosocial adaptations, such as increased physical activity, enhanced self-efficacy, and reduced anxiety and depression, thereby further strengthening functional status and overall quality of life (39).

The results of these studies have vital clinical implications for practice and patient care, demonstrating the consistently positive outcomes of the PR program as rehabilitative interventions. PR has the potential to enhance exercise endurance, minimize impairments, relieve symptoms such as dyspnea, and optimize quality of life and improve overall wellbeing. Collectively, the findings of the current systematic review support PR as a promising and adaptable intervention for individuals with PTLD. While the current body of evidence is limited by heterogeneity in study design, small sample sizes, and a lack of large-scale RCTs, the consistency of improvements in functional capacity and quality of life underscores its clinical value. Future research should focus on standardizing PR protocols, evaluating long-term outcomes, and determining the optimal delivery mode, particularly in resource-limited settings where the burden of disease is challenging. Enhancing the evidence base will be critical for facilitating the integration of PR into routine post-tuberculosis care and ensuring that recovery extends beyond physiological cure to meaningful functional and quality-of-life improvements for patients. While these findings collectively support PR as a valuable intervention for PTLD, several methodological limitations across the included studies must be acknowledged when interpreting the evidence base and planning future research.

## Limitations

Although the existing literature provides valuable insights, several limitations should be acknowledged. First, many studies included relatively small sample sizes, which may limit the generalizability of the findings. Second, substantial heterogeneity in pulmonary rehabilitation protocols and outcome measures across studies complicated direct comparisons. For example, intervention duration ranged from 2 to 24 weeks, rehabilitation settings varied (hospital-, home-, and community-based) and intervention components differed with some studies incorporating inspiratory muscle training while others did not. Similarly, different instruments such as SGRQ and the SF-36, were used to assess quality of life, making cross-study synthesis more challenging. These inconsistencies prevented the identification of an optimal PR protocol or the estimation of standardized treatment effects. Furthermore, the predominance of non-randomized and before-after study designs increased the likelihood of bias and limits the strength of the available evidence. Variations in the definition and diagnostic criteria of post-tuberculosis lung disease also affected the consistency and comparability of findings. For example, some studies included only patients with specific spirometric abnormalities, whereas others used broader clinical diagnostic criteria. Consequently, it remains uncertain whether PR provides similar benefits across the full spectrum of PTLD phenotypes including predominantly obstructive and restrictive ventilatory impairments. A formal risk-of-bias assessment was performed using the Cochrane Risk of Bias 2 (RoB) tool for randomized controlled trials and the ROBINS-1 tool for non-randomized studies. Although the RCTs generally demonstrated a low risk of bias or some concerns, the majority of the non-RCTs studies were having serious overall risk of bias, primarily because of confounding and the absence of concurrent control groups. Therefore, the overall findings should be interpreted with appropriate caution despite the consistent direction of benefit observed across the included studies. In addition, several studies lacked comprehensive statistical reporting and many did not include long-term follow-up, limiting the ability to evaluate the durability of pulmonary rehabilitation benefits. Further research should include larger, well designed randomized controlled trials using standardized PR protocols, consistent PTLD diagnostic criteria, validated outcome measures and longer follow-up periods. Furthermore, studies evaluating the cost-effectiveness and feasibility of implementing PR across different healthcare settings and geographic regions would strengthen the evidence base and support wider clinical implementation.

## Conclusion

This systematic review provides the first comprehensive synthesis of evidence on PR for PTLD, demonstrating that PR interventions can improve exercise capacity and quality of life, though pulmonary function outcomes remain variable. These findings highlight the need for standardized protocols and larger trials to strengthen the evidence base. These interventions have demonstrated improvements in exercise capacity, overall wellbeing, relief from breathing difficulties, and showed variable effects on pulmonary function, with significant improvements in 6 of 13 studies, suggesting that benefits may be limited by irreversible structural damage. Despite heterogeneity in study designs and intervention strategies, the consistent positive outcomes highlight the importance of incorporating PR into the standard care of patients with PTLD.

## Data Availability

The original contributions presented in the study are included in the article/Supplementary material, further inquiries can be directed to the corresponding authors.
