# Supplementary material for: Pulmonary rehabilitation in post-tuberculosis lung disease: a systematic review
Source: Front Med (Lausanne). 2026 Aug 19;13:1929171. doi: 10.3389/fmed.2026.1929171 (PMC13533969; doi:10.3389/fmed.2026.1929171)
Supplement: Supplementary file 1 [file Supplementary_file_1.docx]

| **Study** | **Terminology Used** | **Abbreviation** |
| --- | --- | --- |
| *Tada et al., 2002* | Sequelae of pulmonary tuberculosis / Pulmonary tuberculosis sequelae | — |
| *Ando et al., 2003* | Post-tuberculosis lung disorder | Post-TBC lung disorder |
| *Yoshida et al., 2006* | Pulmonary tuberculosis sequelae | PTS |
| *Rivera et al., 2015* | Sequelae of pulmonary TB | — |
| *Jones et al., 2017* | Post-tuberculosis lung disorder | p-TBLD |
| *Singh et al., 2017* | Chronic lung impairment from previously treated tuberculosis | CLIPTB |
| *Ahmed et al., 2020* | Post-tubercular sequelae / Pulmonary impairment after tuberculosis | PIAT |
| *Tolba et al., 2021* | Treated pulmonary tuberculosis / cured pulmonary TB | PTB (treated) |
| *Fernandez et al., 2022* | Post-pulmonary tuberculosis bronchiectasis | — |
| *Orooj et al., 2023* | Post-pulmonary tuberculosis (treated patients) | Post-PTB |
| *Hussain et al., 2023* | Post-tuberculosis sequelae (with fibrosis- and bronchiectasis-predominant subtypes) | PTB-sequelae |
| *Silva et al., 2025* | Post-tuberculosis lung disease | PTLD |
| *Mtei et al., 2025* | Post-TB lung disease | PTLD |
| *Sinha et al., 2025* | Post tuberculosis lung disease | P-TBLD |
| *Fonseca et al., 2025* | Post-tuberculosis lung disease (people with PTLD) | PTLD / pwPTLD |
| *Torres et al., 2025* | Post-tuberculosis lung disease | PTLD |
| *Rifat et al., 2026* | Post-tuberculosis lung disease | PTLD |

# **Supplementary Table S1.** Terminology Used to Describe Post-Tuberculosis Lung Disease Across Included Studies
